# Richardson’s law and the origins of alcohol research

**DOI:** 10.1073/pnas.2318863122

**Published:** 2025-04-07

**Authors:** Snigdha Mukerjee, Cody A. Siciliano

**Affiliations:** ^a^Department of Pharmacology, Vanderbilt Brain Institute, Vanderbilt Center for Addiction Research, Vanderbilt University, Nashville, TN 37232

**Keywords:** scientific law, Benjamin Ward Richardson, straight-chain monohydroxy alcohols, narcotic actions, ethanol

## Abstract

Interest in the biological actions of alcohols, ethanol in particular, dates back to the earliest historical texts. Alcohol research is now a highly active field with roots in physiology, pharmacology, toxicology, and neuroscience. But at what point did interest and speculation evolve into bona fide science? Here, we set out to identify the earliest systematic empirical investigations into the biological actions of alcohols and unearthed a surprisingly rigorous literature which included a fundamental insight with significant implications for modern research and policy. Through manual backward citation mapping of archived texts, much of which was digitally inaccessible, we outline the origins of alcohol research beginning with a transcribed lecture from Benjamin Ward Richardson in 1869. In the years immediately following, the field of alcohol research was legitimized around what came to be briefly known as “Richardson’s law.” Richardson’s law states that the acute toxicity of straight-chain monohydroxy alcohols is directly proportional to the carbon chain length of the molecule. This law was recognized only briefly not because it was disproven, but rather because it was simply forgotten during the decades that passed between its inception and the advent of systematic bibliographic databases which paved the way for digital archiving. Quantitative analysis of studies spanning a century revealed that across monohydroxy alcohols with one (methanol) to thirteen (tridecanol) carbons, there is a near-deterministic relationship between chain-length and lethal dose (R^2^ = 0.96). Richardson’s law of alcohol potency has silently stood the test of time and is among functional biology’s oldest and least challenged scientific laws.

## Origins of Alcohol Research

Stories are easiest to understand when told from the beginning. In this spirit, as alcohol researchers, we undertook an effort to find the earliest publications investigating the physiological actions of ethanol that would be recognizable as legitimate empirical efforts by today’s standards; in other words, the beginning of modern alcohol research. For many topics in functional biology, literature quickly becomes nonexistent or nonquantitative when looking back beyond the 1940s where MEDLINE indexing became sporadic—this was not the case for alcohol research. A clear network of empirical research was apparent through citation mapping, though exploring the network became precipitously more difficult as we approached the turn of the 20th century. For the works from this era, citations were not linked and in many cases gaining access to full texts meant acquiring hard copies.

Intriguingly, texts in the early 1900s made increasingly frequent reference to the work of one “B.W. Richardson,” as well as to a phenomenon of “Richardson’s law” regarding the toxicity of alcohols. At this point, we became more interested in tracking down the origins of this supposed law than our original goal of finding the origins of the field; ultimately, however, they led to the same end. After mapping citations back to find Richardson’s work, we found that the initial formulation of Richardson’s law was stated in a live experimental demonstration and lecture by Benjamin Ward Richardson in 1869 (1).^*^ Richardson left little room for ambiguity as to where this work should be placed in the timeline of alcohol research: In the opening lines of his lecture, Richardson stated, “I believe I am the first physiologist who has followed the chemists by endeavoring to discover the difference of action of the different [alcohols]”. Further examination of the literature prior to 1869 corroborates Richardson’s observation, though work on the chemical properties of alcohols goes farther back (2, 3), he appears to be the first to systematically apply the scientific method to investigate the biological actions of this family of compounds.

## Benjamin Ward Richardson (1828–1896)

Who was Benjamin Ward Richardson? Despite having apparently been the first researcher to explore the biological actions of alcohols, his name was unknown to us as alcohol researchers. Further investigation revealed that this was not necessarily an oversight on our part; though descriptions of his work in other fields were easy to find, the only reference to alcohol in biographic summaries was to note his views as a staunch prohibitionist. Indeed, his extensive work in many other disciplines was well documented. He is recognized for pioneering contributions to a range of fields, including his work developing many of the first anesthetics and anesthetic delivery systems, his contributions to policy on public hygiene and animal welfare, his work advocating for systematic medical record keeping as well as tracking epidemics, and his role as a driving force in advocating for application of scientific principles in public policy, regulatory standards, and public health in general. He received many accolades for these efforts and was inducted into the Royal College of Physicians as a member in 1856, became a Fellow in 1865, and was inducted as a Fellow of the Royal Society in 1867. In 1893, shortly before his death, he was knighted in recognition of his contributions to humanitarian causes. He appears to be something of a Renaissance man, and in addition to these achievements was also a playwright, inventor, editor, and one of the most prolific writers of his time (4–10).

## Richardson’s Work on Alcohols

Despite these recognitions, his work on alcohol is difficult to find even if one is looking for it directly. To illustrate the degree to which Richardson’s law and his work on alcohol have been lost to the digital world, Benjamin Ward Richardson’s *Wikipedia* page is several pages long, detailing his work on anesthetics and sanitation, his personal life and perspective on alcohol drinking, and even such details as his tenure as the Vice President of the Bread Reform League (11, 12); no mention is made of his establishment of a scientific law, nor that he performed research on alcohol at all. Indeed, not only do summaries of his work make little mention of it, but the few mentions of Richardson’s law in the alcohol literature, in name or in principle, in the last several decades incorrectly cite an unrelated 1867 publication (13), though this is not apparent without access to hardcopy text. Upon obtaining these texts, we found several notable contributions to the alcohol field, which was nonexistent at the time of the publications.

Richardson reported multiple studies on the physiological effects of straight-chain alcohols such as methyl, ethyl, propylic, butyl, amylic, and caproylic alcohols. The naming system is dated as it was before the International Chemical Identifier (InChI) was established to unify the diversity in naming conventions. He emphasized throughout these studies that grouping compounds according to their structure could provide insight into their physiological actions (1, 4, 14, 15); this notion was entirely novel at the time and came well before any theories had been established on structure–function relationships in pharmacology. In his 1869 lecture, addressing contemporary practitioners of medicine, he first shows anatomical dissections of a rabbit after a fatal dose of ethanol to the audience. Next, he brings two guinea pigs to the stage, one dosed with ethanol and the other with pentanol. Despite receiving an equal dose, the former is seemingly unaffected while the latter is completely unresponsive. His conclusions form the basis of what would become Richardson’s law; he states “as the weight of the alcohol increases, as carbon and hydrogen, but especially the carbon, increases, the narcotic action^†^ of the agent is increased. No phenomena can be steadier than these phenomena.” As detailed below, this statement was verified and refined in the following decades, ultimately becoming Richardson’s law.

Richardson’s work with alcohols extends beyond the law, including other basic observations as well as clinical applications. This included the first studies examining the impact of alcohols on body temperature and respiration. Richardson recognized that indiscriminate prescription of spirits as a remedy for common ailments and fever was a mistake and was a strong advocate against it (1, 6). In search of legitimate therapeutic actions, he undertook experiments to substitute functional groups and thereby alter the effects of various alcohols and related compounds (16, 17). He also created qualitative descriptions to classify alcohols’ effects into four distinct “stages of intoxication”: exhilaration, excitement, rambling insensibility, and unconsciousness. This framework allowed him to compare effects across alcohols, as well as characterize the dose dependence of these effects across species. In addition to the cardiovascular, respiratory, and behavioral effects of alcohols, his work was also the first to examine the impact of these compounds on the brain and mind (1, 18, 19). From his works, it becomes clear that he was first and foremost a clinician and relied on his experimental skills to invent therapeutic advances and establish better clinical practices.

Richardson’s work was meticulous and rigorous and would be admirable in any era of science including modern day. For example, for each of the alcohols that he worked with, his papers include a table listing each chemical formula, vapor density, specific gravity, and boiling point. While this might seem like trivial data on its face, obtaining pure and correctly identified alcohols was an issue that caused considerable confusion in the literature until at least the 1900s (1, 20). Further, even upon reading a century and a half later, there is no ambiguity as to which alcohols he was referring to by amylic and caproylic, for example. This second issue—that of a consistent nomenclature for the alcohols—remains an unsolved problem (*SI Appendix*, Table S1). In fact, many publications in the field currently only refer to “alcohol” without specifying the exact compound used. While in many cases it is apparent which alcohol is being referred to in context, we became acutely aware of the difficulties that can result when attempting to search across studies, as the use of common phrasing renders keyword searches ineffective. Because of changes in common usage over time, even when reading in context, had Richardson not stated clear chemical characteristics we would be left to guess which alcohols had been used in these experiments.

## Evaluating Richardson’s Law

Few findings have ever risen to the level of being considered a law in physiology. Fewer still are truly quantitative in nature. To have established such a law but misplaced it in the literature for more than a century is, to our knowledge, unprecedented. Given the historical and scientific weight of this possibility, we sought to first critically evaluate whether Richardson’s claims have proven empirically reliable.

The precise criteria that must be met to constitute a scientific law are debatable, and the term is often applied differently across disciplines; thus, we will first operationally define a scientific law. Operationally defining what constitutes a law is important beyond just semantics, as establishing scientific laws permeates beyond the field and often conveys scientific legitimacy to the public, which in turn influences policy and funding. For example, most subfields of physics allow laws which have many demonstrated violations, including mutually exclusive laws which are allowed to exist together (e.g., Newtonian and quantum physics), and as such, the field of physics has established more laws than any other discipline. Likewise, physics is viewed as the most fundamental, or “hard,” science. Most fields of biology on the other hand allow laws to be struck down by single instances in which they do not hold true, and likewise are admonished for establishing very few, if any, scientific laws. Our operational definition, taken from multiple prior works (21–23), is as follows: A scientific law is a descriptive generalization about how some aspect of the natural world behaves under stated circumstances. A law must be created based on repeated experimental observations that describe a range of natural phenomena. Unlike mathematical descriptions, scientific laws do not express absolute certainty but must predict the outcome of a set of conditions across a wide range of scenarios. While a scientific law predicts the results of certain initial conditions, it does not explain why a relationship exists, nor is it stated as a set of discrete facts.

Richardson’s law, as first formulated in his lecture described above and later refined by multiple subsequent works, states that the acute toxicity of straight-chain monohydroxy alcohols is directly proportional to the carbon chain length of the molecule. During his live experimental demonstration, after displaying the pentanol-dosed, deeply anesthetized guinea pig to the audience, he explains that he could “repeat this experiment with butylic alcohol [butanol], but to produce the same extreme effect, I should require to employ about one-fifth more of the fluid; and I could repeat the experiment with caproylic alcohol [hexanol], but to produce the same effect, I need not take so much of the fluid by one-fifth”(1). In other words, he claims that with each additional carbon molecule added to the alcohol, it requires only 80% of the dosage to produce the same effect. We next sought to empirically determine the degree to which Richardson’s claim has stood the test of time.

**Fig. 1.**
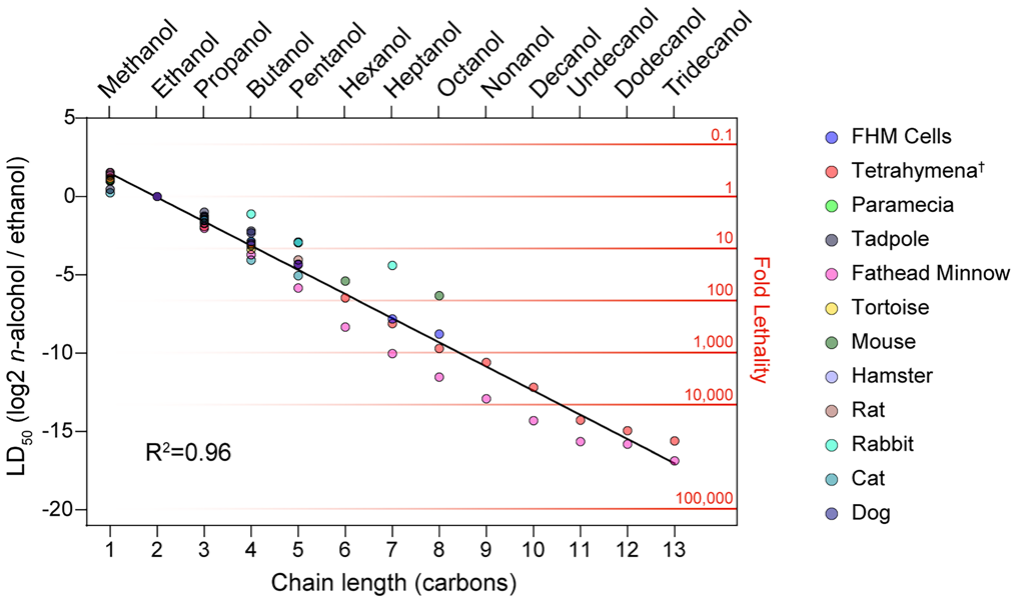
Relative lethality of straight-chain monohydroxy alcohols across organisms. To determine the veracity of Richardson’s law, we performed a meta-analysis of relative lethality of alcohols across a century of studies representing twelve model organisms. Values indicate a fold-change relative to ethanol in the dose at which there is a 50 percent probability of lethality (LD_50_) for each of the straight chain monohydroxy alcohols, from 1 to 13 carbon chain lengths. Values greater than zero indicate lower toxicity than ethanol, while values less than zero indicate higher toxicity, producing lethality at a lower concentration relative to ethanol. None of Richardson’s data were included in the analysis; the data represent studies from 1911 to 2005 (20, 24–31), see *SI Appendix*, *Supplemental Methods*. FHM, fathead minnow. ^†^Values indicate half-maximal inhibitory growth constant [IGC_50_].

A meta-analysis of the literature in relation to Richardson’s law is visualized in Fig. 1. The half-maximal lethal dose (LD_50_) for each of the straight-chain monohydroxy alcohols from 1 to 13 carbon chain lengths (methanol to tridecanol) is plotted across 12 organisms (20, 24–31) (*SI Appendix*, *Supplemental Methods*). The rapid decrease in LD_50_ shows that, indeed, the acute biological toxicity of monohydroxy alcohols is directly proportional to the carbon chain length of the molecule. Despite spanning a century of literature and testing toxicity in a range of scenarios, from single-cell organisms to mammals, Richardson’s law accounts for the results with impressive accuracy: More than 95% of the variance was explained by a linear regression with a slope of −1.54 (R^2^ = 0.96). Thus, the addition of each carbon atom reduces the lethal dose by a fold-change of 0.344 [2^−1.54^]; said another way, each additional carbon makes the alcohol 291% as potent at inducing death than the last.

By the early 1900s, there was clear consensus in the literature that Richardson’s statements regarding the relative toxicity of monohydroxy alcohols had proved universal and accurate to the point of constituting a scientific law (24, 32–35). Several important discoveries stemmed from Richardson’s law, some of which have been recognized as a law in their own right, including the Meyer–Overton rule.^‡^ If Richardson’s law stood the test of time, why then has it faded so completely from the literature? While we can only speculate, several factors likely played a part. One potentially contributing factor was Richardson’s stance on the use of ethanol in medicine and society, which called for “total abstinence” (19, 36–38)—a position which was unpopular among many scientists and doctors at the time (9, 39).

**Fig. 2.**
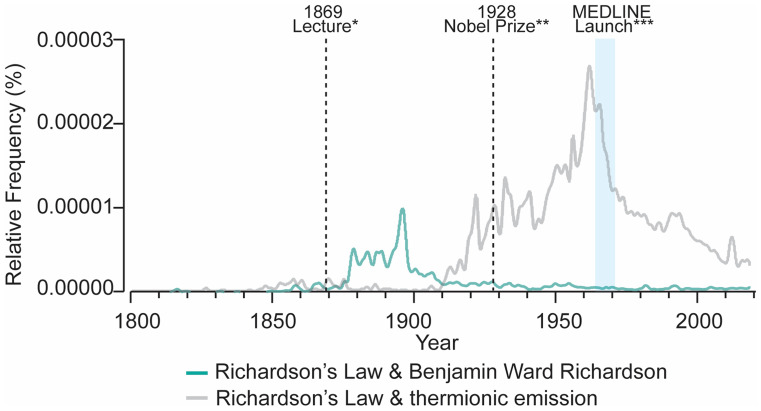
Rise and fall of Richardson’s law visualized via N-gram analysis. To visualize the prevalence of the phrase “Richardson’s law” over time, we utilized Google N-gram viewer, which allows for querying frequency of phrase usage in over 8 million texts published between 1800 and 2019. We hypothesized that the advent of the thermionic emission law in physics, which was also called Richardson’s law, may have contributed to the paucity of literature on Benjamin Ward Richardson’s work. Indeed, querying Richardson’s law together with either Benjamin Ward Richardson or thermionic emission supports this notion. See *SI Appendix*, *Supplemental Methods* for exact queries and parameters. *In 1869, Richardson’s law of alcohol potency was introduced in a pivotal lecture by Benjamin Ward Richardson entitled, “Physiological research on alcohols.” **A contributing event that likely caused Richardson’s law of alcohol potency to be overlooked in the literature, was the awarding of the 1928 Nobel Prize in Physics to Owen Willans Richardson for establishing the thermionic emission law, which was also dubbed “Richardson’s law” by the Nobel Prize Committee. ***Relative proximity to the advent of MEDLINE indexing in the 1960s, when digital archiving began in earnest, likely contributed to the predominance of Richardson’s thermionic emission law in digital literature.

A likely major contributing factor was an unfortunate nomenclature oversight, whereby the 1928 Nobel Prize in Physics, awarded to Owen Willans Richardson for his work on the thermionic emission law, was also dubbed Richardson’s law by the Nobel Prize Committee (40). The timing of the second, unrelated “Richardson’s law” likely played a role in the disproportionate representation on modern day search engines, as the 1920s and 30s saw massive increases in systematic archiving and scientific journal circulation. By the 1940s, the first systematic indexing efforts were underway. These early precursors would soon lead to the MEDLINE/PubMed indexing in the 1960s, which remains predominant in today’s literature. Analysis of usage of the phrase “Richardson’s law” in the literature over time corroborates these hypotheses (Fig. 2).

Going forward, in an attempt to disambiguate the two laws, we recommend that the law based on Owen Willans Richardson’s work be referred to as the “thermionic emission law.” However, the literature cannot be undone; thus, we also recommend that the law based on Benjamin Ward Richardson’s work be referred to as “Richardson’s law of alcohol potency.”

## Refining the Law and Reconciling Methanol Toxicity

It is important to address the acute toxicity of methanol in the context of Richardson’s law of alcohol potency, which has been pointed out as a potential violation (41). Indeed, methanol, often referred to as “wood alcohol,” is infamous for causing sickness and blindness when consumed directly or as a contaminant in ethanol sources (42). Methanol has a single carbon and is the lightest of the straight-chain alcohols. Methanol poisoning can lead to optic nerve damage, however, this is caused by its metabolites, formaldehyde, and formic acid (42, 43). While the mechanism of methanol toxicity was not appreciated until many decades later, Richardson explicitly separated this type of secondary toxicity even in his initial claims and studies. In his 1869 lecture, after detailing the recent discovery by August Wilhelm von Hofmann that methanol when oxidized becomes formaldehyde (44)^§^ and just prior to beginning his experimental demonstration, he states that discussion would be limited to “postmortem results in cases where death is a direct result. Changes in structure from the slow actions of alcohol I must reserve for a future lecture”. This statement thus excluded the actions of secondary metabolites, as in the case of methanol’s prodrug actions through formaldehyde. Later, as the number of alcohols in the chemical family continued to expand, the law was further refined to straight-chain monohydroxy alcohols specifically. This was based on the observation that the isoalcohols, like isopropyl or isobutyl alcohol, are less toxic than their straight chain counterparts regardless of chain length (20, 24, 34). Together, the final formulation thus specifies the *acute* toxicity of *straight-chain monohydroxy* alcohols.

It is difficult to overstate the degree of foresight with which Richardson interpreted and reported his results. To put this into context, consider that pharmacokinetics had yet to be conceived of, and drug metabolism in the human body was still decades away from being a fringe concept. The idea that the structure of a compound might be predictive of its biological function was also decades ahead of his time. Indeed, though he had put forth the idea of structure–function relationships for alcohols and chemical compounds in general the concept had yet to gain traction. He hoped that “by proving physiological action and the relation of chemical constitution to physiological action” it would eventually lead to reform, stating that he was “certain the time must soon come when the books we call Phamacopœias will be everywhere reconstructed on this basis of thought, and when the chemist and Physician will become one and one” (14). On top of formulating his law and his conviction that chemical structure determines pharmacodynamics, to have foreseen that drugs might be metabolized by the body to produce secondary actions was visionary.

## Potential Mechanisms and Related Findings

As discussed above, to meet the criteria for a scientific law, there need not be mechanistic or explanatory reasoning; nonetheless, a near-deterministic association between two variables always begs the question of “why?”. Richardson speculated as to the mechanism of death in his experiments, noting that, in the case of ethanol, “the final act rests with the heart: The heart continues to beat when the breathing has ceased”. He also noted several postmortem indications that could be observed across the alcohols, including congestion of the kidneys and that the brain was suffused with blood. Other indications were specific to the long-chain alcohols, including muscular tremors which presented prior to death. He described these tremors as being identical to delirium tremens in humans, a condition that can be triggered by severe withdrawal in heavy drinkers.

While the mechanism for chain-length-dependent toxicity is not entirely understood, the leading theory posits that this relationship results from increased membrane-disrupting properties of the alcohol. As more carbons are added to the straight-chain, alcohols become less polar because the nonpolar hydrocarbon chain makes up a larger part of the molecule. This means that they are more soluble in fats and oils (hydrophobic) and less soluble in water. This increased hydrophobicity allows long-chain alcohols to more easily enter cell membranes and disrupt the lipid bilayer (45, 46).

More recent work directly measured the effects of various alcohols on membrane fluidity/rigidity, demonstrating chain-length dependence of the membrane-disrupting properties of these compounds. Indeed, pioneering work by Dora Goldstein in the 1970s and 80s built directly on Richardson’s law of alcohol potency in principle, though it is not mentioned by name. Her work not only directly examined the chain-length-dependent impact of alcohols on membrane characteristics in brain tissue but also linked these actions to the intoxicating effects of ethanol as well as ethanol dependence and withdrawal (47–49).

In addition to the membrane fluidity hypotheses above, it remains possible that the chain-length-dependent potency of alcohols could be the result of a protein/receptor target, through canonical structure–function-based pharmacodynamics. Most of the work in this area has been focused on ethanol, but to date, there has not been a definitive receptor target identified. Nonetheless, despite a lack of an identified binding site, ethanol is known to modulate the function of a number of receptors and ion channels [see (50) for review.] Going forward, it may be informative to determine the degree to which the synaptic actions of ethanol are recapitulated by longer chain alcohols, and whether these effects are also beholden to Richardson’s law of alcohol potency.

## Implications of Richardson’s Law for Modern Research and Policy

In addition to historical importance, Richardson’s law of alcohol potency has implications for ongoing alcohol research as well as regulatory policy. Regarding ongoing research, several fundamental aspects of ethanol’s biological actions, such as identification of the primary site of action that mediates ethanol reward and reinforcement, remain unsolved. While Richardson’s law does not speak to these outstanding questions directly, it is important to realize that most laws in science are not powerful because of their immediate applicability, but rather for their reliability. Even small statements can be very powerful building blocks when they can be assumed as facts. The applicability of structure–function relationships is perhaps more so than at any point in the past given the advent of computational approaches, such as virtual ligand screening, which can turn structure–function relationships into foundational discoveries. For example, searching for targets which display a chain-length-dependent change in affinity across the alcohols with a similar slope as is seen with the LD_50_ of these compounds could reveal the underlying mechanisms. Intriguingly, alcohol effects on dopamine neuron firing, which are thought to play a role in mediating ethanol reinforcement, are also chain-length dependent (51) and insight may be gained in this area by leveraging knowledge of these structure–function relationships.

Regarding implications for regulatory standards and policy, Richardson’s law of alcohol potency may inform an important issue that Richardson repeatedly raised directly in his writing and lectures: purity standards of distilled alcohols. Regulatory standards for permissible levels of alcohols in commercial ethanol are essential for safeguarding public health, ensuring product quality, and supporting the responsible use of ethanol. While acceptable methanol concentrations in commercial ethanol beverages are regulated [maximums vary by spirit but most are capped at 7 g of methanol per liter of ethanol (52, 53)], there are no regulatory standards for maximum allowable concentrations of long-chain alcohols in the United States, and it is unclear if the presence of these compounds is directly monitored by distillers or government agencies.^¶^ The few studies that have reported concentrations of long-chain alcohols in commercial ethanol have found a wide range depending on distillation method/type of beverage, but it is clear that many contain significant amounts (54, 55).

Although safety of chronic exposure to each of the alcohols is not fully established, Richardson’s law of alcohol potency provides a guideline that can be used to extrapolate reasonable limits. Based on the assumption that fold-change in lethality approximates fold-change in reasonable acceptable limits, we propose that allowable concentrations of alcohols in commercial beverages without labeling should be limited to a maximum of 86 mM multiplied by 0.344 for each additional carbon. The 86 mM constant is derived from the maximum allowable ethanol concentration in nonalcoholic beverages in the United States (0.5% ABV^#^) (56, 57), and the 0.344 multiplier is derived from the meta-analysis above indicating a log2 fold change of −1.54 per carbon atom [$\log_{2}\left(0.344\right)=-1.54]$. Thus, maximum allowable concentrations, without labeling, for monohydroxy alcohols with three through thirteen carbons would be as follows (mM, % v/v): propanol (29.58, 0.22), butanol (10.18, 0.09), pentanol (3.5, 0.038), hexanol (1.2, 0.015), heptanol (0.41, 0.0059), octanol (0.14, 0.0023), nonanol (0.049, 0.00086), decanol (0.0168, 0.00032), undecanol (0.0058, 0.00012), dodecanol (0.001996, 0.00004), tridecanol (0.000687, 0.00001). This would then limit the lethality of the solution to the same level as would be expected from the maximum allowable concentration of ethanol in nonalcoholic beverages (e.g., 0.5% ethanol and 0.038% pentanol ABV solutions are equi-lethal according to the analysis above).

## Conclusions

In summary, we mapped citations back from the modern literature to the mid-1800s, ultimately pinpointing the origins of biological research into the alcohols. In doing so, we “discovered” surprisingly insightful reports, including the establishment of Richardson’s law of alcohol potency. In addition to its inherent importance from a meta-scientific perspective, the topic raises a discussion which is more relevant than ever to today’s scientists: How many discoveries with profound implications for ongoing research have been lost to time, either left behind in the annals of hardcopy texts or buried in the ever-expanding digital literature? The vital importance of preserving historical insights by digitally archiving early scientific works, buried in years of research that has become lost to the field, is particularly pertinent as computational synthesis of literature gains prominence in contemporary science.

## Supplementary Material

Appendix 01 (PDF)

## Data Availability

All study data are included in the article and/or *SI Appendix*. Previously published data were used for this work (20, 24–31).

## Footnotes

^*^We were unable to ascertain the exact location of the lecture, which was delivered on Tuesday December 7th 1869, as part of a series of lectures on a range of topics which Richardson gave monthly that year entitled ‘Lectures on Experimental and Practical Medicine’. Published in the *Medical Times and Gazette* the lectures appear to have taken place at various locations throughout London, UK., including several at the Royal Polytechnic Institute (present-day University of Westminster) (58).

^†^In modern parlance, “narcotic action” equates roughly with anesthetic and sedative/hypnotic actions.

^‡^Established independently by Hans Horst Meyer and Charles Ernest Overton in 1899 and 1901, respectively, the Meyer-Overton rule states that anesthetic potency is dictated by the partition coefficient of the compound.

^§^Hofmann’s discoveries dealt only with chemical properties, it was not appreciated until decades later that drugs were metabolized in the body.

^¶^We attempted to gain clarification on this issue by contacting the Bureau of Alcohol, Tobacco, and Firearms and were informed that regulatory standards for commercial ethanol are the purview of the Alcohol and Tobacco Tax and Trade Bureau (TTB); however, the TTB did not respond to several inquiries.

^#^We assume equivalence between ABV and % v/v, and do not account for possible volume changes during mixing.
