# Supplementary material for: Richardson’s law and the origins of alcohol research
Source: Proc Natl Acad Sci U S A. 2025 Apr 7;122(15):e2318863122. doi: 10.1073/pnas.2318863122 (PMC12012554; doi:10.1073/pnas.2318863122)
Supplement: Supplementary file 1 — Appendix 01 (PDF) [file pnas.2318863122.sapp.pdf]

PNAS

Supporting Information for
Richardson's Law and the Origins of Alcohol Research

Snigdha Mukerjee, Cody A. Siciliano*

Department of Pharmacology, Vanderbilt Brain Institute, Vanderbilt Center for Addiction Research, Vanderbilt University, Nashville, TN 37232, USA.

* Corresponding Author:
Cody A. Siciliano, PhD
2222 Peirce Avenue
467 Robinson Research Building
Nashville, TN 37232
Email: Cody.Siciliano@Vanderbilt.Edu

This PDF file includes:

Methods associated with Figures 1 and 2
Supplemental Table 1

Supporting Information Text

Supplemental methods

Meta-analysis of alcohol toxicity by chain length

Data in Figure 1 were compiled from published tables and text, (1–8) and mined from The Registry of Toxic Effects of Chemical Substances (RTECS) database(9). Care was taken to find studies reporting lethality of multiple alcohols using matched methodologies. Only studies which reported ethanol and at least two other alcohols using the same methodology were included; this allowed for values to be normalized within study, with ethanol as the reference point, thereby converting each into a common unit framework (relative lethality) despite many different methods and routes of administration being used across studies. Accordingly, within each study, LD_{50} values were normalized as $[n - alcohol\ LD_{50}\ (M)\ \ /\ ethanol\ LD_{50}\ (M)]$. In cases where dosage was reported by weight or by volume, values were first converted to moles. Specific gravities and molecular weights used to convert the values are listed in Supplemental Table 1 for methanol through decanol. For undecanol, dodecanol, and tridecanol, molecular weights of 172.31, 186.33, 200.36 g/mol and specific gravities of 0.83, 0.83, and 0.846 were assumed, respectively. In cases where the same species was reported in multiple studies, values were averaged after normalization. Mouse was represented in two studies, and rat and rabbit were each represented in three studies; values for all other organisms were derived from one study.

Study inclusion was based on three criteria: 1) studies which reported lethality of ethanol and at least two other monohydroxy alcohols using identical methodology in the same organism(s), 2) that lethality was reported as an LD_{50}, and 3) that values were reported in molar or that sufficient detail of the dosing was provided to allow for conversion. Studies that met all three criteria but only reported toxicity in repeated dosing/chronic exposure paradigms were excluded. Studies authored by Benjamin Ward Richardson and studies examining alcohol effects on bacteria were also excluded.

Temporal analysis of n-grams related to Richardson's laws

To visualize the frequency of published word sequences (n-grams) related to Richardson's Laws (i.e. Richardson's law of alcohol potency and Richardson's law of thermionic emission) we used Google Books Ngram Viewer(10, 11) which allows for querying n-grams across more than 8 million texts published from 1800 to 2019 (http://books.google.com/ngrams). The prompts used to generate the data in Figure 2 were "(Richardson's law +Benjamin Ward Richardson)" and "(Richardson's law +thermionic emission)", entered without quotation marks. The two prompts were entered as separate queries to allow for case-insensitive searching and the parameters were set to the following: smoothing = 0, timescale: 1800 – 2019 years, case insensitive enabled. The data were extracted with PlotDigitizer and replotted in GraphPad Prism v10 (see Figure 2). Data was scraped from queries made on June 15th, 2024.

	Structure	Formula	Molecular Weight (g/mol)	Boiling Point (°F at 760 mmHg)	Specific Gravity (at 68 °F, *unless indicated)	Alternate Names
Methanol	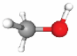	CH_4O	32.04	148.3°	0.792	Methyl alcohol<br>Carbinol<br>Wood alcohol
Ethanol	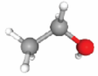	C_2H_6O	46.07	173.3°	0.790	Alcohol<br>Ethyl alcohol<br>Grain alcohol<br>Methylcarbinol
Propanol	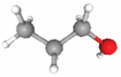	C_3H_8O	60.10	207.0°	0.803	Propyl alcohol<br>1-propanol
Butanol	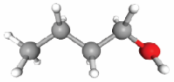	$C_4H_{10}O$	74.12	243.9°	0.810	Butyl alcohol<br>1-butanol
Pentanol	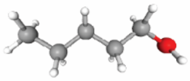	$C_5H_{12}O$	88.15	280.0°	0.818*	Amyl alcohol<br>Pentyl alohol<br>Amylol<br>1-pentanol
Hexanol	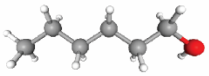	$C_6H_{14}O$	102.17	314.8°	0.850	Caproyl alcohol<br>Amylcarbinol<br>Hexyl alcohol<br>1-hexanol
Heptanol	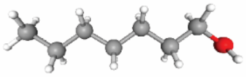	$C_7H_{16}O$	116.20	349.0°	0.822	Gentanol<br>Enanthyl alcohol<br>Heptyl alcohol<br>1-heptanol
Octanol	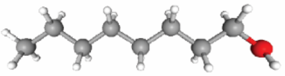	$C_8H_{18}O$	130.23	383.0°	0.829	Caprylic alcohol<br>Octyl alcohol<br>1-ocatanol
Nonanol	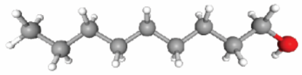	$C_9H_{20}O$	144.25	415.0°	0.827	Nonyl alcohol<br>Pelargonic acid<br>Octyl carbinol<br>1-nonanol
Decanol	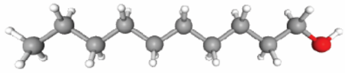	$C_{10}H_{22}O$	158.28	446.0°	0.840	Caprinic alcohol<br>Decyl alcohol<br>Nonylcarbinol<br>1-decanol

Table 1. Properties of monohydroxy alcohols by chain-length. The molecular weight and boiling point of primary alcohols increases with every carbon atom that is added to the straight-chain molecule of monohydroxy alcohol. The difference in boiling point is exploited during distillation to attempt to separate the alcohols. Each of the alcohols is referred to by multiple names in the modern literature and usage differs over time; a fully standardized nomenclature has not been delineated, complicating communication and evaluation of the literature. The top alternative names are listed here, but there are many more for each alcohol. Values, alternative names, and 3D structures were taken from PubChem.

SI References

1. V. Oettingen, W. Felix, *The Aliphatic Alcohols: their Toxieity and Potential Dangers in relation to their Chemical Constitution and their Fate in Metabolism* (1943).

2. I. Macht, A toxicological study of some alcohols, with especial reference to isomers. *The Journal of Pharmacological and Experimental Therapeutics* **16**, 1–10 (1920).

3. T. W. Schultz, L. M. Arnold, T. S. Wilke, M. P. Moulton, Relationships of quantitative structure-activity for normal aliphatic alcohols. *Ecotoxicol. Environ. Saf.* **19**, 247–253 (1990).

4. J. C. Munch, E. W. Schwartze, Narcotic and toxic potency of aliphatic alcohols upon rabbits. *J. Lab. Clin. Med.* **10**, 985–996 (1925).

5. C. E. Bills, A PHARMACOLOGICAL COMPARISON OF SIX ALCOHOLS, SINGLY AND IN ADMIXTURE, ON PARAMECIUM. *J. Pharmacol. Exp. Ther.* **22**, 49–57 (1923).

6. J. C. Brandão, H. H. L. Bohets, I. E. Van De Vyver, P. J. Dierickx, Correlation between the in vitro cytotoxicity to cultured fathead minnow fish cells and fish lethality data for 50 chemicals. *Chemosphere* **25**, 553–562 (1992).

7. M. Tichý, V. Trcka, Z. Roth, M. Krivucová, QSAR analysis and data extrapolation among mammals in a series of aliphatic alcohols. *Environ. Health Perspect.* **61**, 321–328 (1985).

8. H. M. Vernon, The action of homologous alcohols and aldehydes on the tortoise heart. *J. Physiol.* **43**, 325–342 (1911).

9. National Institute for Occupational Safety and Health, The Registry of Toxic Effects of Chemical Substances (RTECS). Deposited 2005.

10. J.-B. Michel, *et al.*, Quantitative analysis of culture using millions of digitized books. *Science* **331**, 176–182 (2011).

11. Y. Lin, *et al.*, Syntactic Annotations for the Google Books NGram Corpus. *Annual Meeting of the Association for Computational Linguistics* (2012).
